# Low *REST* Expression Indicates a Biomarker of Poor Prognosis in Patients with Renal Cell Carcinoma

**DOI:** 10.1155/2021/6682758

**Published:** 2021-03-24

**Authors:** Chaoxiang Lv, Yuanguo Li, Qiqi Zhang, Yanyan Chen, Dandan Wei, Tiecheng Wang, Bo Zhou

**Affiliations:** ^1^The Key Laboratory of Molecular Epigenetic, Institute of Genetics and Cytology, Northeast Normal University, Changchun 130024, China; ^2^Key Laboratory of JiLin Province for Zoonosis Prevention and Control, Institute of Military Veterinary Medicine, Academy of Military Medical Sciences, Changchun 130122, China; ^3^College of Animal Medicine, Jilin University, Changchun 130000, China; ^4^College of Basic Medicine, Changchun University of Chinese Medicine, Changchun 130117, China

## Abstract

It was initially found that neural-restrictive silencer factor/repressor 1-silencing transcription factor (*REST*) is a transcriptional repressor of neuronal genes in nonneuronal cells. However, it is reported to be abundantly expressed in various types of aggressive cancer cells. In this study, we evaluated the expression patterns of *REST* in renal cell carcinoma and found that its expression is lower in tumor tissues compared to normal tissues. The chi-square test showed that the low *REST* expression was closely related to patients' clinicopathologic parameters, including the pathologic stage and survival status. ROC curve showed that *REST* had excellent clinical diagnostic prospect. In addition, patients with low *REST* expression had poor over survival (OS) and relapse-free survival (RFS). Univariate and multivariate Cox regression analysis confirmed that the low *REST* expression was an independent predictor of poor prognosis in renal cell carcinoma. Gene set enrichment analysis identified P53 pathway, reactive oxygen species pathway, glycolysis, DNA repair, cholesterol homeostasis, and MYC targets V2 enriched with low *REST* expression phenotype. These results suggested that *REST* may be a novel biomarker for the diagnosis and prognosis of renal cell carcinoma in clinical applications.

## 1. Introduction

Renal cell carcinoma (KIRC), a common urinary system tumor, accounts for 2% to 3% of human malignant tumors [1–3]. It has been reported that 90 percent of patients had been diagnosed with KIRC [4, 5]. In many countries, the incidence and case fatality of KIRC are steadily increasing [6, 7]. Although the significant progress had been made in diagnosis and treatment, the patient's prognosis is still worse. In recent years, with the further research in tumor molecular biology, targeted therapy has become a new diagnosis and treatment strategy in current clinical applications [8]. Therefore, the search for new molecular targets is extremely important for the clinical diagnosis, treatment, and prognostic monitoring of KIRC.

RE 1-silencing transcription factor (*REST*), also known as neural-restrictive silencing factor (NRSF), is a zinc-finger transcription factor that inhibits target gene transcription by recruiting transcription coinhibitors such as histone deacetylase (HDACs) [9–11]. Moreover, *REST* can serve as a hub for the recruitment of multiple chromatin-modifying enzymes, revealing the interdependencies between enzymes that influence gene regulation [12]. In addition, *REST* inhibits the expression of neuroendocrine-related genes during neuronal differentiation [13–15]. As a result, *REST* was initially regarded as the primary regulator of neurogenesis. Recent studies have reported that *REST* can inhibit the occurrence of tumors, and *REST* gene deletion or mutation is closely related to the occurrence of many tumors such as small-cell lung cancer [16], prostate cancer [17], and ovarian cancer [18].

In the current study, our group focused on the relationship between the *REST* expression and clinicopathological features, diagnostic value, and prognostic assessment of patients with KIRC. We compared the *REST* mRNA expression between cancer patients and healthy individuals and analyzed the application prospect and diagnostic significance of the *REST* expression in KIRC. In addition, we investigated the association between the *REST* expression and the clinicopathologic features, including OS and RFS. The results revealed that the *REST* expression is an independent risk factor for poor survival, suggesting that *REST* may be a valuable diagnostic and prognostic biomarker for KIRC.

## 2. Materials and Methods

### 2.1. Dataset Mining and Database Collection

We first obtained RNAseq of *REST* and clinical information of KIRC patients from The Cancer Genome Atlas (TCGA) dataset. RNAseq was converted to RSEM by estimating the log2 (*x* + 1) normalized counts which are used for subsequent analysis by selecting *R* software (version 4.0.1) [19].

### 2.2. Data Analysis

The data was analyzed by the program package in the *R* software. The box plot showed the mRNA expression of *REST* in the KIRC dataset through ggplot2 visual analysis. The chi-square test was used to evaluate the correlation between the *REST* expression and clinical characteristics of KIRC patients. The receiver operating characteristic (ROC) curve was used to evaluate the diagnostic value of expression through the pROC software. Kaplan-Meier survival curves were performed to compare OS and RFS in different groups of patients. Risk regression models were used to perform univariate and multivariate analysis to evaluate the prognostic value of the *REST* expression. *P* < 0.05 is considered statistically significant.

### 2.3. Gene Set Enrichment Analysis (GSEA)

In order to detect the distribution of predefined genomes and determine the potential mechanism to influence the effect of the *REST* expression on the prognosis of KIRC patients, we opted for GSEA (version 4.0.3). This analysis was performed through the “h.all.v7.2.symbols.gmt” gene set in the Molecular Signatures database [20]. Gene sets with a normal *P* value <0.05 were regarded as significantly enriched.

## 3. Results

### 3.1. The Patient Clinical Characteristics and Expression of REST in KIRC

**Figure 1:**
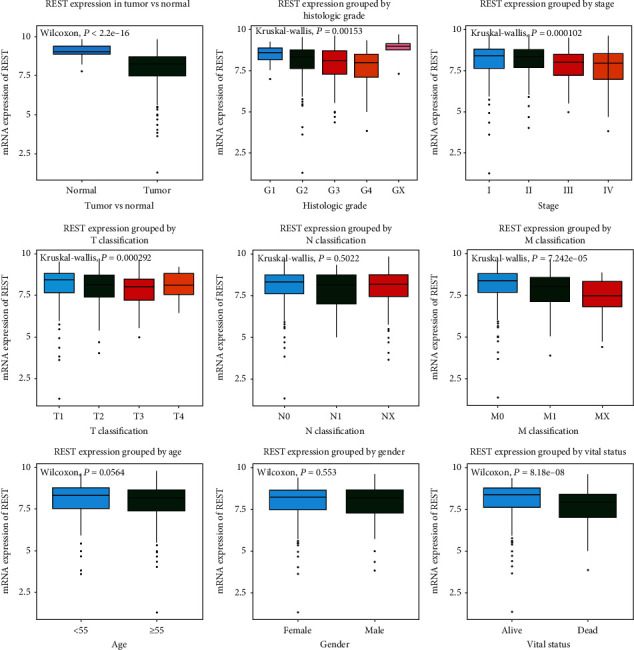
Expression pattern of *REST* in KIRC. Expression of *REST* between tumor and normal tissue was compared. The expression of *REST* was compared according to different histologic grade, stage, T/N/M classification, as well as age, gender and vital status.

Through using *R* software, clinical data of 373 patients were collected from the TCGA database, including the patient's age, gender, histological type, histologic grade, histologic stage, and TNM classification, as well as radiation therapy, residual tumor, vital status, and relapse-free survival (Table 1). Subsequently, we analyzed the expression pattern of *REST*. As shown in Figure 1, *REST* was significantly higher in normal tissues than tumor tissues (*P* = 2.20 × 10^−16^), which indicated that the expression of *REST* is downregulated in KIRC. Additionally, differences in the *REST* expression were observed according to patient histological grade (*P* = 0.00153), pathologic stage (*P* = 0.000102), T classification (*P* = 0.000292), N classification (*P* = 0.0000724), and vital status (*P* = 8.18 × 10^−8^).

### 3.2. The Diagnostic Significance of the REST Expression and Relationship between Clinical Characteristics in KIRC

**Figure 2:**
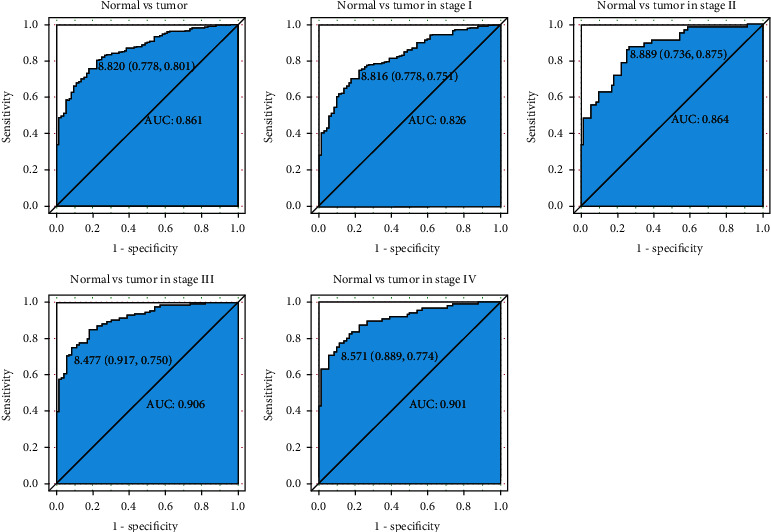
Diagnosis value of *REST* expression in KIRC. The ROC curves of *REST* expression in cancerous vs. normal tissues was generated. Cancerous vs. normal liver tissues was analyzed in different stages of KIRC.

We previously showed that the *REST* expression was significantly downregulated in KIRC. To evaluate the diagnostic significance of the *REST* expression, ROC curve was established. We found that the *REST* expression had excellent diagnostic value overall (AUC = 0.861; Figure 2). Subsequently, we analyzed the diagnostic value of the *REST* expression in different stages of KIRC, including stage I cancer (AUC = 0.826), stage II cancer (AUC = 0.864), stage III cancer (AUC = 0.906), and stage IV (AUC = 0.901). Subsequently, we divided patients into two groups (high expression and low expression) according to the ROC curve threshold (Figure 2(a)). As shown in Table 2, the low *REST* expression was significantly associated with patient age (*P* = 0.00500), histologic grade (*P* = 0.0260), pathologic stage (*P* = 0.0140), T classification (*P* = 0.0260), M classification (*P* = 0.00400), and vital status (*P* = 0.000).

### 3.3. The Effect of the Low REST Expression for OS in Patients with KIRC

**Figure 3:**
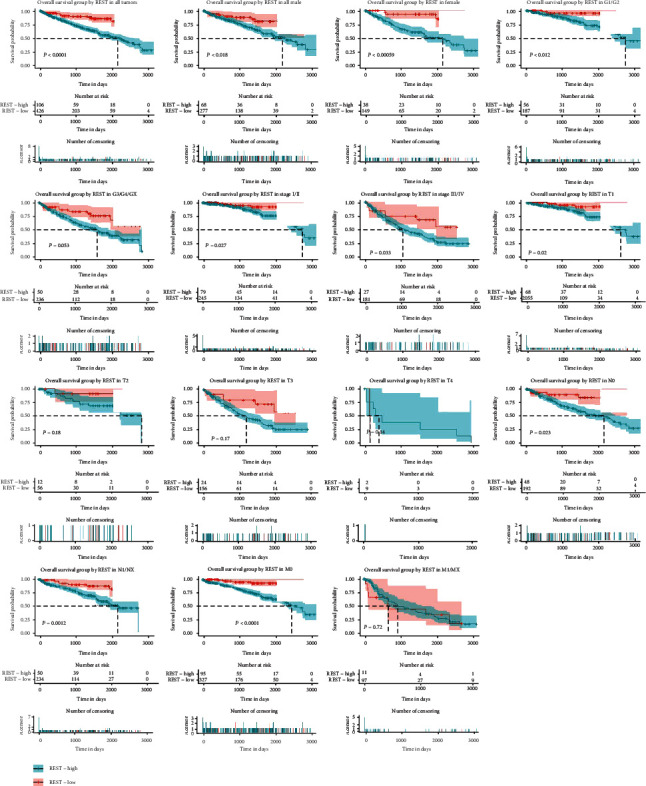
The effect of the *REST* expression on OS in KIRC. Kaplan-Meier curves of the *REST* expression in all patients. Kaplan-Meier curves of the *REST* expression in the subgroup.

We used survival analysis to evaluate the effect of the *REST* expression on the over survival (OS) of kidney cancer patients. As shown in Figure 3, Kaplan-Meier survival curves shown that the low *REST* expression significantly decreased the patient's OS (*P* < 0.000100). In addition, we also observed that male patients with low *REST* expression had a shorter OS (*P* = 0.0180), and female patients (*P* = 0.000590). Subgroup analysis found that the low *REST* expression significantly affects patient OS in G1/G2 (*P* = 0.0120), G3/G4/GX (*P* = 0.00530), stage I/II (*P* = 0.027), stage III/IV (*P* = 0.0330), T1 (*P* = 0.0200), T3 (*P* = 0.0170), N0 (*P* = 0.0230), N1/NX (*P* = 0.00120), and M0 (*P* < 0.000100). Subsequently, we selected potential variables that were significant in univariate analysis to conduct multivariable Cox analysis (Table 3). We found that low *REST* is an independent risk factor for poor OS in patients with KIRC (hazard ratio [HR] = 1.20, 95% confidence interval [CI]: 1.04–1.39, *P* = 0.0100).

### 3.4. The Effect of the Low REST Expression for RFS in Patients with KIRC

**Figure 4:**
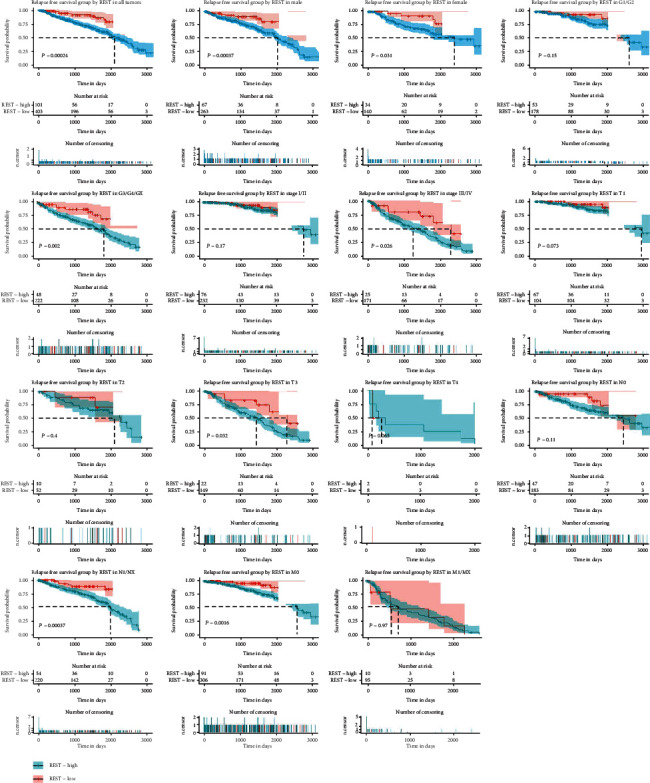
The effect of the *REST* expression on RFS in KIRC. Kaplan-Meier curves of the *REST* expression in all patients. Kaplan-Meier curves of the *REST* expression in the subgroup.

We have previously shown that the low *REST* expression predicts a poor prognosis for OS among KIRC patients. To assess the correlation between the *REST* expression and patients' relapse-free survival (RFS), the Kaplan-Meier database was performed. As shown in Figure 4, Kaplan-Meier survival curves shown that the low *REST* expression significantly decreased the patient's RFS (*P* = 0.000240). In addition, we also observed that male patients with low *REST* expression had shorter RFS (*P* = 0.00370) and female patient (*P* = 0.0310). Subgroup analysis found that the low *REST* expression significantly affects patient RFS in G3/G4/GX (*P* = 0.00200), stage III/IV (*P* = 0.0260), T3 (*P* = 0.0320), N1/NX (*P* = 0.000370), and M0 (*P* = 0.00160). Subsequently, we selected potential variables that were significant in univariate analysis to conduct multivariable Cox analysis (Table 4). We found that low *REST* is an independent risk factor for poor RFS in kidney cancer patients (hazard ratio [HR] = 1.21, 95% confidence interval [CI]: 1.04–1.41, *P* = 0.0140).

### 3.5. Low REST Expression-Related Signaling Pathway

**Figure 5:**
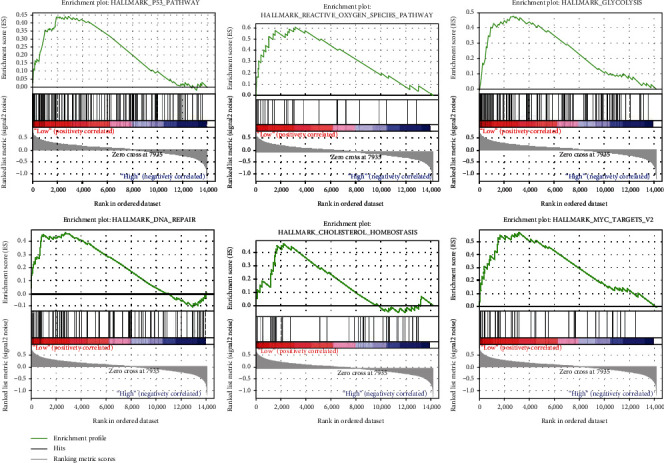
Gene set enrichment plots. GSEA results showing differential enrichment of genes related to P53 pathway, reactive oxygen species pathway, glycolysis, DNA repair, cholesterol homeostasis, and MYC targets V2 in KIRC cases with low *REST* expression.

Identifying the activation of signaling pathways will help to better understand the interactions, reactions, and relationships between molecules [20, 21]. To determine the signaling pathways activated in KIRC, we used GSEA to analyze the low and high *REST* expression datasets. The results showed that P53 pathway, reactive oxygen species pathway, glycolysis, DNA repair, cholesterol homeostasis, and MYC targets V2 were all enriched to the low *REST* expression phenotype (Table 5, Figure 5).

## 4. Discussion

By analyzing the TCGA-KIRC dataset, we observed that *REST* was low expressed in KIRC, and its expression gradually decreased with patients' higher historical level and tumor level. In addition, our results showed that the low expression of *REST* is negatively correlated with patient survival. Through the survival curve, we found that KIRC patients with low *REST* expression had poor OS and RFS. Univariate and multivariate Cox regression analysis confirmed that *REST* was an independent predictor of poor prognosis among KIRC patients.

Previous studies have reported that *REST* is highly expressed in a variety of tumors, including glioma, neuroblastoma, and medulloblastoma [22–24]. However, the expression of *REST* in KIRC has been rarely reported. In this study, we observed that the *REST* expression is low in cancerous tissues, which contradicts other findings about the *REST* expression in tumors, suggesting that the *REST* expression is complex in tumors. Interestingly, we also found that the *REST* expression gradually downregulated as histologic grade increasing from G1 to G4, as histologic stage increased from I to IV and as T classification increased from T1 to T3. The reason for the slightly higher expression in patients with GX and T4 is unclear, but this may be due to the limited samples from advanced cancer.

*REST* is a key target oncogenic transformation and neural differentiation and inhibits transcription by regulating chromatin structure or inhibiting underlying transcription mechanisms [25–27]. During neuron development, *REST* is the main transcriptional repressor of neuron-specific genes and plays an important role in nonneuron and neuronal progenitor cells through histone deacetylation, chromatin remodeling, methylation, and other mechanisms [28–31]. Recent studies have confirmed that *REST* is closely related to carcinogenesis and cancer progression [32]. In this study, we observed that the low *REST* expression gradually decreased with the increase of degree of malignant tumor, which indicated that *REST* may be an important regulatory gene for tumor occurrence and development. In addition, ROC curve analysis provided evidence that *REST* can be developed as a biomarker for the diagnosis of KIRC.

Although the association of *REST* with various cancer types has been reported, the mechanism by which *REST* plays a role in cancer progression and tumorigenesis is still unclear. Studies have verified that decreased *REST* expression promotes epithelial cell transformation [33]. In ovarian cancer, *REST* regulates the growth and survival of tumor cells via the regulation of mTOR signaling [34]. In addition, the *REST* expression is closely related to the depth of malignant tumor invasion, TNM stage, and local lymph node metastasis, and the patients with high *REST* expression had a worse overall survival in medulloblastoma [35]. These indicate that *REST* can be used as a drug target and a new prognostic factor for medulloblastoma. In contrast, our findings suggest that the *REST* expression in kidney cancer patients is associated with patient OS and RFS. These data suggested that *REST* may serve as a potential marker for adjuvant diagnosis, efficacy, and prognosis assessment of KIRC.

To our knowledge, this is the first report on the correlation between *REST* expression and clinical features and prognosis prediction in KIRC patients based on the TCGA database. Our study revealed that *REST* had good clinical diagnostic value and is an independent risk factor for poor prognosis in KIRC patients. However, in the future, the structural network and specific mechanism between *REST* downregulation and shortened survival time of kidney cancer patients still need to be improved, so as to provide better treatment strategies for KIRC patients.

## Figures and Tables

**Table 1:** The clinical characteristics of patients in the present study.

Parameters	Variables	Numbers (%)
Age	≥55	362 (67.92)
	<55	171 (32.08)
Gender	Male	345 (64.73)
	Female	188(35.27
Histologic grade	NA	3(0.56)
	G1	14(2.63)
	G2	230 (43.15)
	G3	205 (38.46)
	G4	76 (14.26)
	GX	5(0.94)
Pathologic stage	I	269 (50.47)
	II	56 (10.51)
	III	124 (23.26)
	IV	84 (15.76)
M classification	NA	2(0.38)
	M0	422 (79.17)
	M1	79 (14.82)
	MX	30(5.63)
N classification	N0	240 (45.03)
	N1	17 (3.19)
	NX	276(51.78)
T classification	T1	274 (51.41)
	T2	68 (12.76)
	T3	180 (33.77)
	T4	11 (2.06)
Vital status	Dead	372 (69.79)
	Survival	161 (30.21)
Relapse	NA	28(5.25)
	NO	362(67.92)
	YES	143(26.83)
CREBBP	High	106(19.87)
	Low	427(80.13)

NA: not available.

**Table 2:** Associations between the clinicopathologic variables and *REST* expression.

Parameters	Variables	Numbers	*REST*				*X* ^2^	*P* value
			High	Prop (%)	Low	Prop (%)		
Age	≥55	362	60	56.60	302	70.73	7.773	0.005
	<55	171	46	43.40	125	29.27		
Gender	Male	345	68	64.15	277	64.87	0.0193	0.890
	Female	188	38	35.85	150	35.13		
Histologic grade	G1	14	4	3.77	10	2.36	11.006	0.026
	G2	230	52	49.06	178	41.98		
	G3	205	39	36.79	166	39.15		
	G4	76	8	7.55	68	16.04		
	GX	5	3	2.83	2	0.47		
Pathologic stage	I	269	67	63.21	202	47.31	10.566	0.014
	II	56	12	11.32	44	10.30		
	III	124	16	15.09	108	25.29		
	IV	84	11	10.38	73	17.10		
M classification	M0	422	95	89.62	327	76.94	11.002	0.004
	M1	79	11	10.38	68	16		
	MX	30	0	0	30	7.06		
N classification	N0	240	48	45.28	192	44.97	0.7340	0.693
	N1	17	2	1.89	15	3.51		
	NX	276	56	52.83	220	51.52		
T classification	T1	274	68	64.15	206	48.24	9.267	0.026
	T2	68	12	11.32	56	13.11		
	T3	180	24	22.64	156	36.54		
	T4	11	2	1.89	9	2.11		
Vital status	Dead	161	13	12.26	148	34.66	20.204	0.0001
	Survival	372	93	87.74	279	65.34		

**Table 3:** Univariate and multivariate analysis of over survival in patients with KIRC.

	Univariate analysis			Multivariate analysis		
	Hazard ratio	CI95	*P* value	Hazard ratio	CI95	*P* value
Age	1.89	1.30-2.75	0.001	1.53	1.03-2.26	0.033
Gender	1.04	0.75-1.43	0.826			
Histologic grade	2.06	1.71-2.47	0.0001	1.56	1.25-1.94	0.0001
Pathologic stage	1.96	1.71-2.24	0.0001	2.04	1.40-2.97	0.0001
M classification	2.47	1.94-3.19	0.0001	0.82	0.50-1.35	0.431
N classification	0.86	10.7-1.01	0.063			
T classification	2.07	1.74-2.46	0.0001	0.80	0.55-1.18	0.260
*REST*	1.30	1.15-1.46	0.0001	1.20	1.04-1.39	0.010

**Table 4:** Univariate and multivariate analysis of relapse-free survival in patients with KIRC.

	Univariate analysis			Multivariate analysis		
	Hazard ratio	CI95	*P* value	Hazard ratio	CI95	*P* value
Age	1.33	0.93-1.91	0.117			
Gender	0.77	0.54-1.10	0.155			
Histologic grade	1.97	1.62-2.38	0.0001	1.31	1.04-1.64	0.020
Pathologic stage	2.42	2.07-2.83	0.0001	2.62	1.84-3.80	0.0001
M classification	3.42	2.69-4.34	0.0001	1.08	0.66-1.78	0.762
N classification	1.03	0.87-1.22	0.730			
T classification	2.34	1.94-2.83	0.0001	0.73	0.51-1.05	0.092
*REST*	1.34	1.18-1.52	0.0001	1.21	1.04-1.41	0.014

**Table 5:** Gene set enrichment analysis in phenotype low among KIRC.

Name	ES	NES	NOM *P* value
HALLMARK_P53_PATHWAY	0.44	1.82	0.000
HALLMARK_REACTIVE_OXYGEN_SPECIES_PATHWAY	0.61	1.70	0.007
HALLMARK_GLYCOLYSIS	0.48	1.65	0.033
HALLMARK_DNA_REPAIR	0.47	1.64	0.028
HALLMARK_CHOLESTEROL_HOMEOSTASIS	0.47	1.61	0.019
HALLMARK_MYC_TARGETS_V2	0.57	1.55	0.041

ES: Enrichment score; NES: normalized enrichment score; NOM: nominal.

## Data Availability

TCGA-KIRC dataset used to support the findings of this study are available from the corresponding author upon request.
